# Radial Peripapillary Capillary Plexus Perfusion and Endothelial Dysfunction in Early Post-SARS-CoV-2 Infection

**DOI:** 10.3390/vision6020026

**Published:** 2022-05-16

**Authors:** Maria Cristina Savastano, Luca Santoro, Emanuele Crincoli, Claudia Fossataro, Gloria Gambini, Alfonso Savastano, Umberto De Vico, Angelo Santoliquido, Antonio Nesci, Francesco Landi, Stanislao Rizzo, on behalf of Gemelli against COVID-19 Post-Acute Care Study Group

**Affiliations:** 1 Ophthalmology Unit, Fondazione Policlinico Universitario A. Gemelli IRCCS, 00118-00199 Rome, Italy; mariacristina.savastano@gmail.com (M.C.S.); emanuelecrincoli1@gmail.com (E.C.); gambini.gloria@gmail.com (G.G.); alfonso.savastano@policlinicogemelli.it (A.S.); umbertodevico@gmail.com (U.D.V.); stanislao.rizzo@policlinicogemelli.it (S.R.); 2 Department of Ophthalmology, Catholic University “Sacro Cuore”, 00118-00199 Rome, Italy; 3 Department of Internal Medicine, Catholic University of Rome, 00118-00199 Rome, Italy; luca.santoro@policlinicogemelli.it; 4 Department of Cardiovascular Sciences, Fondazione Policlinico Universitario A. Gemelli IRCCS, Università Cattolica del Sacro Cuore School of Medicine, 00118-00199 Rome, Italy; angelo.santoliquido@unicatt.it (A.S.); antonio.nesci@unicatt.it (A.N.); 5 Division of Geriatrics, Fondazione Policlinico Universitario A. Gemelli IRCCS, Università Cattolica del Sacro Cuore School of Medicine, 00118-00199 Rome, Italy; francesco.landi@unicatt.it; 6 Consiglio Nazionale delle Ricerche, Istituto di Neuroscienze, 56121-56128 Pisa, Italy

**Keywords:** COVID-19, endothelial dysfunction, OCT angiography, retinal vascular layers, macula, SARS-CoV-2, flow-mediated dilation, diabetes

## Abstract

Background: Endothelial cells damage and thromboinflammation are considered key elements in the generation of organ impairment in patients with COVID-19 disease. The endothelial function is evaluated by measuring flow-mediated dilation (FMD). We aimed to analyze the association between FMD impairment and retinal vascular parameters in early post-COVID-19 patients. 00118-00199Tomography (OCT), OCT Angiography (OCTA) and slit lamp examination were performed. FMD ≤ 7% was considered as pathological. Our primary outcome was to assess potential differences in the radial peripapillary capillary plexus flow index (RPCP-FI) and RPCP density (RPCP-D) values between post-COVID-19 patients with and without FMD impairment. The associations of other retinal vascular parameters with FMD impairment were assessed as secondary endpoints. Results: FMD impairment was detected in 31 patients (37.8%). RPCP-FI (*p* = 0.047), age (*p* = 0.048) and prevalence of diabetes (*p* = 0.046) significantly differed in patients with FMD ≤ 7% in regression analysis. RPCP-FI was linearly correlated with FMD values (R = 0.244, *p* =0.027). SCT was found to be lower in patients with impaired FMD (*p* = 0.004), although this difference was only a trend in binary logistic regression output (*p* = 0.07). Conclusions: Early post-COVID-19 patients showed a higher prevalence of FMD impairment compared to the general population. Age, diabetes and RPCP-FI were independently correlated with the presence of endothelial impairment in the early post-infective period.

## 1. Introduction

At the end of 2019, an outbreak of pneumonia with unknown cause began in China’s Hubei Province, raising a global health concern due to its ease of transmission. A severe acute respiratory syndrome coronavirus 2, named SARS-CoV-2, was identified as the pathogen responsible for the disease, which Chinese scientists named coronavirus-19 (COVID-19) [1]. Since then, the virus has spread all over the world, causing the death of 6 million people to the present date [2]. As the body requires time to respond to viral antigens, symptoms may appear 2 to 14 days after exposure to the virus [3]. Despite being asymptomatic in 30–55% of individuals with positive real-time polymerase chain reaction (RT-PCR) nasopharyngeal swab results, the disease may manifest with flu-like symptoms, and a small percentage of individuals (about 10% of all symptomatic patients) can present dyspnea, severe interstitial pneumonia, acute respiratory distress syndrome (ARDS), thromboembolic events and multiorgan dysfunction [4]. In particular, thromboembolic events are among the leading causes of mortality and morbidity in patients with COVID-19 disease, manifesting as deep vein thrombosis (DVT), pulmonary embolism (PE), microvascular thrombosis, cerebrovascular and cardiac disease [5]. Autoptic findings in COVID-19 patients have demonstrated extensive microvascular injury, and endothelial dysfunction has been recognized to play an important role in the pathogenesis and clinical course of the disease [6,7].

The most widely used and validated technique to measure endothelial function is based on brachial artery reactivity: flow-mediated dilation (FMD), assessed with this technique, measures the nitric oxide-mediated vasodilation produced by increased flow after a period of ischemia (endothelium-dependent vasodilation) [8,9]. Impaired FMD has been associated with conditions predisposing to atherosclerosis and cardiovascular disease, representing an early step in the development of subclinical target organ damages and late clinical events [10]. Several studies demonstrated the prognostic value of FMD for cardiovascular (CV) events: a meta-analysis indicated a significant 8–13% lower risk of CV events per percent point increase in FMD, a reduction which is relevant both in high- and in low-risk populations [11]. Endothelium damage and thromboinflammation are considered key elements in the generation of extra-pulmonary organ damage in patients with COVID-19 disease, including hepatocellular damage, acute kidney injury and myocardial infarction [12]. Moreover, recent research has showed how endothelium disfunction, evaluated with the FMD technique, is related to a more serious development of COVID-19 disease. [13] Additionally, recent findings suggest that the vascular supply of the eye could be compromised by the infection, causing ophthalmic sequelae, a finding yet to be understood. In particular, the retinal vasculature has been shown to present specific anomalies in vessel density and vascular dilation in COVID-19 patients in comparison to the healthy population [14,15].

Thus, the purpose of the present study was to correlate retinal vascular parameters, analyzed using optic coherence tomography (OCT) and OCT angiography (OCTA), to the level of endothelial impairment measured by FMD in early post-COVID-19 patients.

## 2. Materials and Methods

This observational monocentric cohort study was designed and supported by the Gemelli Against COVID-19 Post-Acute Care Study Group as part of a series of studies aimed to clarify the subacute consequences of COVID-19 disease [16]. Recruitment took place at the Fondazione Policlinico Universitario Agostino Gemelli IRCSS from 1 March 2020 to 3 October 2020 and included patients randomly selected from hospital recordings. In particular, the study population included patients whose primary diagnosis of admission was SARS-CoV-2 infection. The patients were enrolled within 1 month after discharge to detect endothelial function impairment caused by the infection. Exclusion criteria were pre-existing documented FMD < 7%, previous myocardial infarction, previous ischemic stroke, peripheral arterial occlusive disease, choroidal atrophy, pathological myopia, exudative age-related macular degeneration (AMD), previous episode of central serous chorioretinopathy or pre-existing documented pachychoroid, choroidal neovascularization, retinal occlusive diseases, glaucoma, acquired and hereditary optic neuropathy, hereditary retinal diseases, demyelinating disorders, neurodegenerative disorders, ongoing chemotherapy and drug abuse [17].

The study was approved by the Catholic University/Fondazione Policlinico Universitario A. Gemelli IRCCS Institutional Ethics Committee (protocol ID number: 003220/20). Each patient signed an informed consent and received a full explanation of the target protocol, in conformity to the declaration of Helsinki. The manuscript was reviewed by all the authors, who guarantee for the reliability of the data and for the respect of the study protocol.

### 2.1. Outcome Measures

The outcome of the study was to detect an association between pathological FMD and OCTA-derived retinal vascular parameters in early post-COVID-19 patients. FMD ≤ 7% was considered pathological, according to the scientific literature [18]. The primary endpoint was to detect a difference in the radial peripapillary capillary plexus flow index (RPCP-FI) and the radial peripapillary capillary plexus density (RPCP-D) mean values in post-COVID-19 patients with and without FMD impairment. Radial peripapillary capillary plexus (RPCP) vascular parameters were chosen as the primary endpoint considering the previously reported finding of impairment in post-COVID-19 patients compared with healthy controls [14]. The associations of other retinal vascular parameters with FMD impairment were taken into account as secondary endpoints. In particular, the analyzed parameters were subfoveal choroidal thickness (SCT), superficial capillary plexus density (SCP-D), superficial capillary plexus perfusion (SCP-P), deep capillary plexus density (DCP-D), deep capillary plexus perfusion (DCP-P), foveal avascular zone (FAZ) area and FAZ perimeter. We did not consider any healthy control group, since the aim of our analysis was to find a significant difference between ex-COVID-19 patients with and without FMD impairment. However, our group recently reported a significant difference in RPCP vascular parameters between ex-COVID-19 patients and healthy controls [13].

### 2.2. Procedures and Instruments

**Figure 1:**
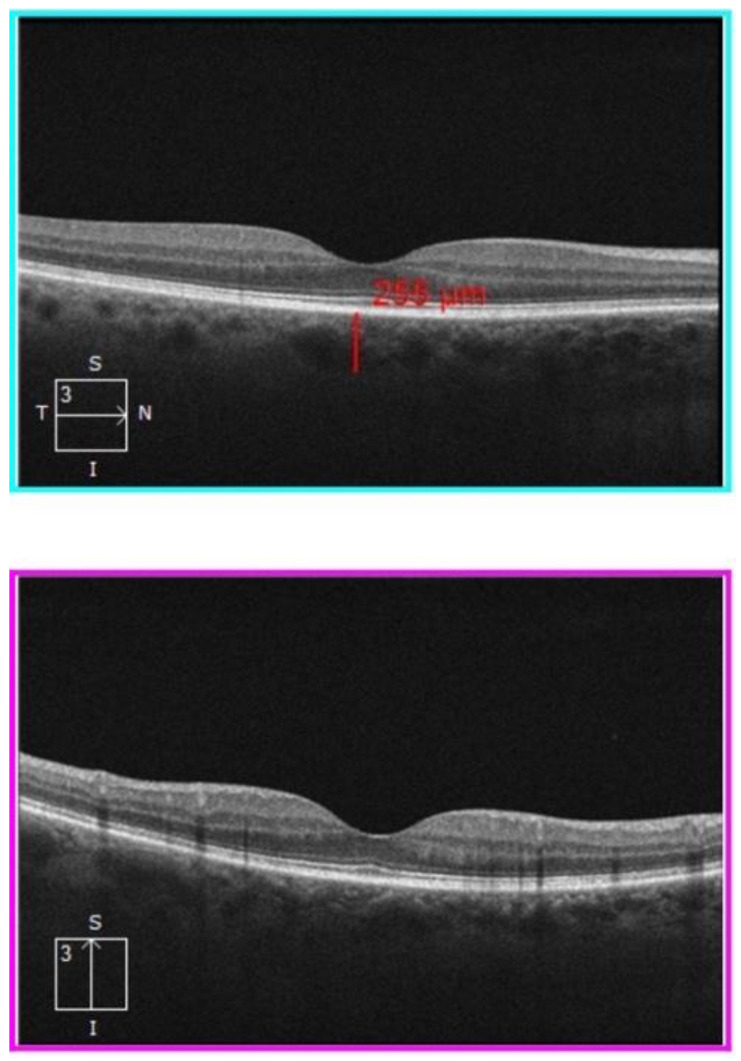
High-resolution 5-line HD scan OCT. Horizontal- and vertical-line OCT B scan. The upper image shows how the SCT was measured by the examiner. SCT = subfoveal choroidal thickness.

**Figure 2:**
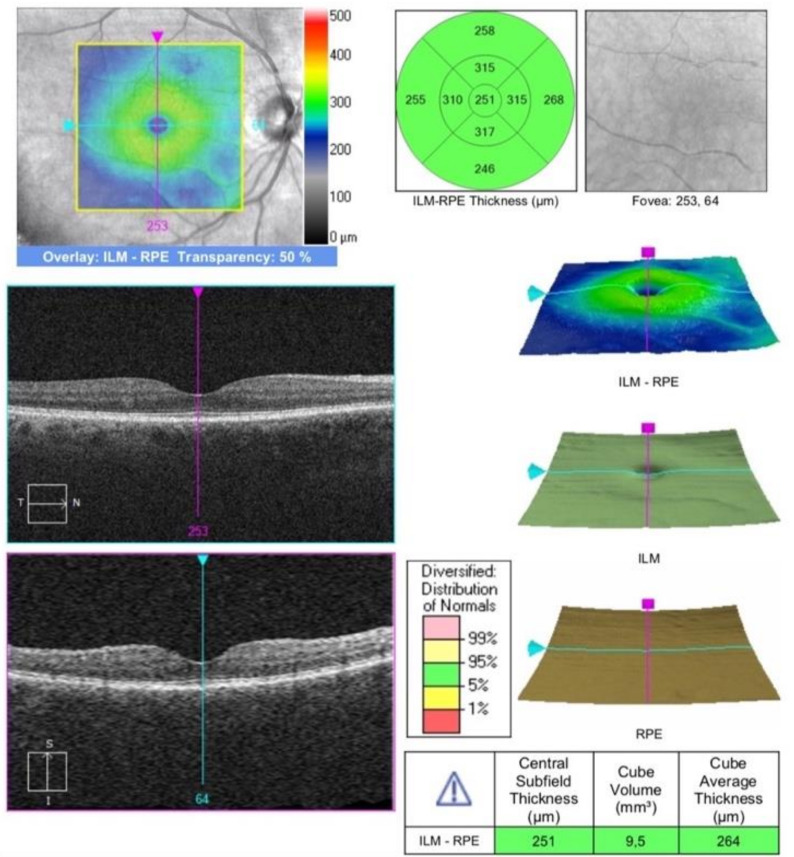
OCT macular cube.

The study protocol included only one clinical evaluation within 1 month after discharge, consisting in FMD assessment and ophthalmologic examination. The latter included best corrected visual acuity (BCVA) assessment, intraocular pressure (IOP) measurement, slit lamp and fundus examination, OCT scan including infrared and angiography sequences. Structural OCT and OCTA analysis were performed by an expert physician using the Spectral Domain Zeiss Cirrus 5000-HD-OCT Angioplex (sw version 10.0, Carl Zeiss, Meditec, Inc., EIDW, Dublin, CA, USA). One eye for each patient was randomly chosen for examination. In case of unilateral eye disease, the other eye was selected. OCT acquisitions were high-resolution 5-line HD scan at posterior pole and macular cube (200 × 200, Figure 1 and Figure 2). Two independent masked examiners manually measured the SCT in the fovea region on cross-sectional OCT B-scans. The measure was calculated from the outside layer of the retinal pigment epithelium (RPE) to the choroid–sclera junction [19]. 

**Figure 3:**
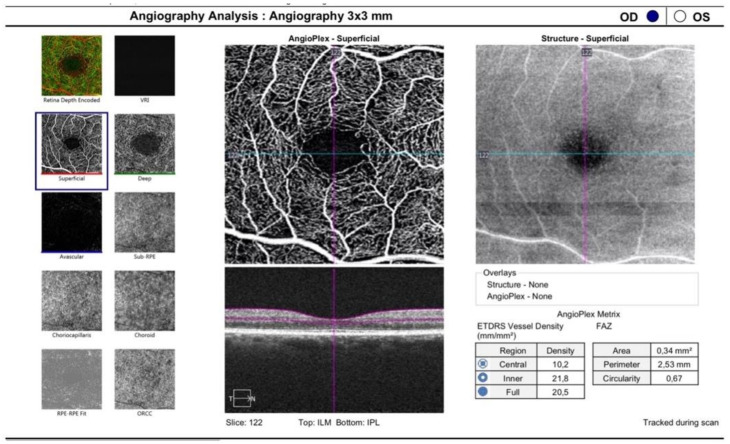
A 3 × 3 mm OCTA scan focused on the fovea. SCP, DCP and CC could be analyzed. SCP = superficial capillary plexus; DCP = deep capillary plexus; CC = choriocapillaris.

The OCTA scans, focused on the fovea, were performed using a 3 × 3 mm volume scan pattern. (Figure 3) An image of the superficial capillary plexus (SCP) and deep capillary plexus (DCP) was automatically generated by the software using automated layer segmentation. A manual correction of the segmentation lines was performed, if necessary. Imaging processing was performed using ImageJ software (National Institutes of Health, Maryland, MD, USA). Vessel Density (VD) was expressed in percentage by taking the ratio of the total vessel area (all pixels with a ratio value between 0.7 and 1.0) to the whole area of the considered region (size of the image in pixels). MATLAB v7.10 (Mathworks, Inc., Natick, MA) was used for vascular density calculation. The FAZ border was independently manually outlined by two trained physicians and expressed in mm^2^ [20].

**Figure 4:**
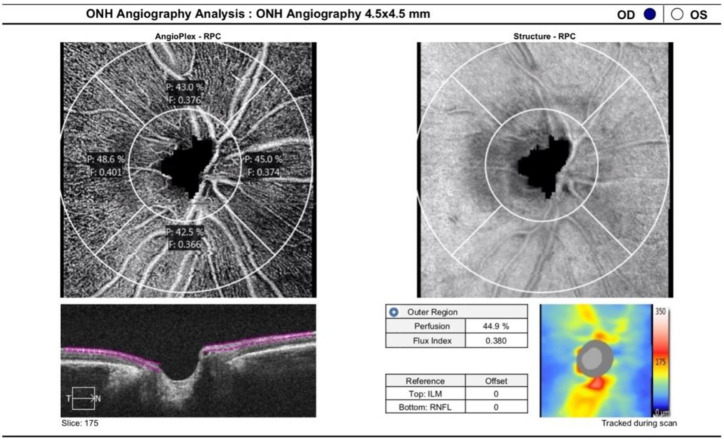
ONH angiography 4.5 × 4.5 mm.

The peripapillary OCTA scan protocol was 4.5 × 4.5 mm acquisition-centered on the optic disc (Figure 4). Two-dimensional en-face OCT angiograms of the radial peripapillary capillary plexus (RPCP) layer were generated with automated segmentation software (Cirrus 10.0), with the RPCP defined as the segment extending superficially from the inner limiting membrane to the posterior surface of the retinal nerve fiber layer (RNFL). En-face images were processed using a customized software with an interactive interface [21]. A combination of a global threshold, Hessian filter, and an adaptive one is the basis of the software in order to obtain a binary vessel maps, on which quantitative indices of blood flow are calculated in MATLAB (R2017a; MathWorks, Inc., Natick, MA, USA). Using the en-face retinal angiogram, the peripapillary flow index was determined as the average decorrelation value in the peripapillary region. To define the peripapillary vessel density, the proportion of the total area occupied by vessels was considered. The vessels were identified as the pixels with decorrelation values over the threshold in the noise region that were two standard deviations higher than the mean decorrelation value. The avascular zone of the optic nerve head (ONH) was manually selected to establish the baseline background noise level for a global threshold, and the ONH was excluded from quantification [22]. Finally, RPCP vessel density (RPCP-VD) and RPCP-FI were recorded and used for analysis.

For the study of the endothelial function, we evaluated the brachial artery reactivity, emerging as the most well-established technique used in adults. FMD assessed with this technique measures the nitric oxide-mediated vasodilation produced by increased flow after a period of ischemia (endothelium-dependent vasodilation). This measurement was performed in accordance with the actual guidelines [8,9]. The patient had to rest supine for 15–20 min, then the right brachial artery was scanned over a longitudinal section 5–7 cm above the antecubital fossa. The arterial diameter was measured from the tunica intima of both anterior and posterior walls and, a pulse Doppler velocity signal was registered. After the first measurement, the blood pressure cuff was inflated in order to reach a blood pressure value of 250 mmHg, which was kept stable for 5 min, then the cuff was suddenly deflated. A second scan was performed continuously for 90 s to evaluate changes into the arterial diameter after reactive hyperemia. A pulse Doppler velocity signal was also recorded within 15 s after the cuff deflation to examine the maximal hyperemic velocity. The FMD data are expressed as percentage increases relative to the baseline diameters. A single skilled examiner, blinded to the subject’s details, executed all ultrasound examinations in a quiet, temperature-controlled room. All scans were performed always at the same time (8.00 am) to avoid the reported circadian variation of endothelial function [23]. All patients avoided any exercise, food and vasoactive substances (e.g., drugs, tobacco, coffee) for at least 12 h before the analysis.

### 2.3. Statistical Analysis

Sample size calculation was performed using G*power (3.1.9.7 software) by setting the desired power of the study to 20%, the alpha error to 5% and a clinically significant difference to 5% in RPCP-FI. SPSS software (IBM SPSS Statistics 26.0) was used to perform the statistical analysis. As concerns quantitative variables, normality of the distribution was evaluated using the Shapiro–Wilk test, and univariate comparison between the two groups was performed using a 2-tailed *t* test for independent groups. Linear correlations were established using Spearman’s Test. Qualitative variables were confronted by means of a X^2^ test or a Fisher exact test when appropriate. The agreement between the two graders in manual measurements was determined through Bland Altman plot analysis. Binary logistic regression was performed to evaluate the actual strength of the associations detected by the univariate analysis as concerns predictors of FMD impairment in early post-COVID-19 patients. The Hoser–Lemeshow test was performed to assess the goodness of fit of the logistic regression. A *p* value < 0.05 was considered statistically significant.

## 3. Results

The study population included 82 Caucasian post-COVID-19 patients with a mean age of 52.9 ±13.5 years; 58.5% of the patients were males. Thirty-six (43.9%) patients were affected by diabetes, 23.2% of the cohort suffered from systemic arterial hypertension, and 21 (25.6%) patients had autoimmune diseases. Chronic kidney disease was present in 9.8% of the population, while cognitive impairment was reported in 7 (8.5%) patients. All patients showed normal carotid or only increased carotid intima–media thickness, in the absence of carotid plaques or stenosis. Mean body mass index (BMI) was 25.7 ± 4.3, with eight patients exceeding a score of 30 (cut off for obesity). Nine (10.9%) patients required intensive care unit (ICU) admission, and 33 (40.2%) patients were treated with oxygen therapy during their hospital stay. As concerns respiratory support, non-invasive mechanical ventilation was provided to 11 patients, and invasive ventilation was necessary for 4 patients. Two cases of pulmonary embolism and two cases of venous thrombosis complicated the clinical course of the infection in four patients. Lastly, 6 patients were treated with anti-aggregation therapy, and heparin was administered to 28 (34.1%) patients during admission. The remaining and the above-mentioned anamnestic and hospital stay data are summarized in Table 1.

An FMD ≤ 7% was detected in 31 (37.8%) patients, while the other patients (51 patients, 60.2%) showed a normal FMD value. Patients with impaired FMD were significantly older than patients with normal FMD (*p* < 0.001) and had a higher prevalence of diabetes (*p* = 0.04) and systemic arterial hypertension (*p* = 0.002). The lower FMD value reported in this group could be explained in light of the observation that a more severe COVID-19 disease developed in elderly patients and in those with other comorbidities. Only the differences in age and prevalence of diabetes were confirmed by subsequent binary logistic regression analysis (respectively, *p* = 0.048 and *p* = 0.046). The Hoser–Lemeshow test confirmed the goodness of fit of the analysis. In particular, diabetic post-COVID-19 patients had a higher probability of endothelial dysfunction, with an odds ratio (OR) of 3.24. None of the remaining anamnestic and clinical course data were found to be significantly different between the two groups (see Table 1).

**Figure 5:**
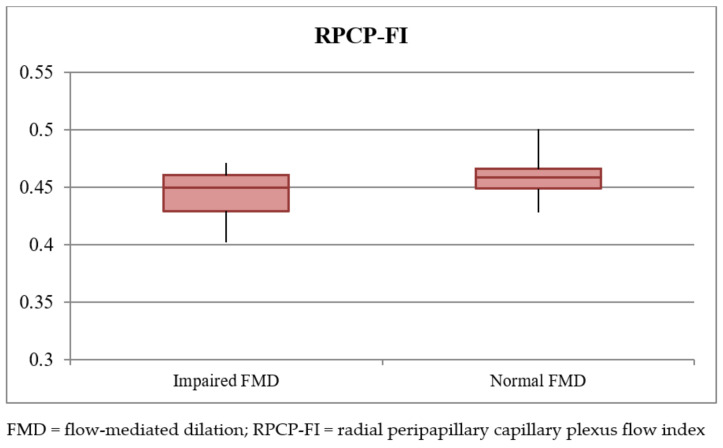
Radial peripapillary capillary plexus flow index (RPCP-FI) values in the impaired (flow-mediated dilation) FMD and normal FMD subgroups. Box plot showing the distribution of the RPCP-FI values in the impaired FMD and normal FMD subgroups.

**Figure 6:**
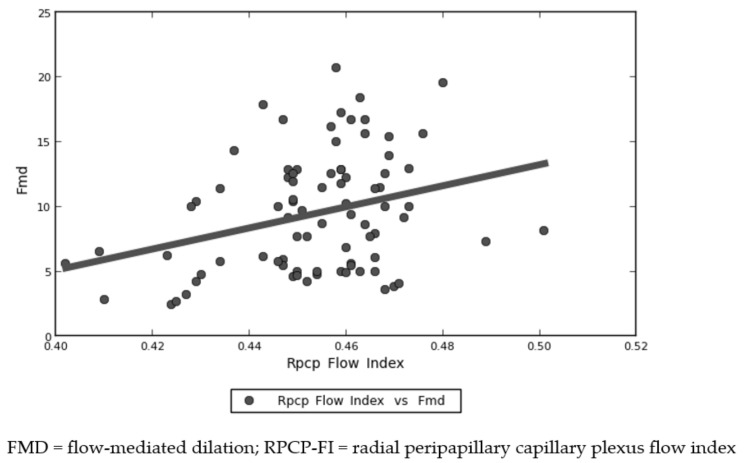
Distribution of radial peripapillary capillary plexus flow index (RPCP-FI) and flow-mediated dilation (FMD) values. Spearman’s linear correlation between RPCP-FI and FMD values in early post-COVID-19 patients.

**Figure 7:**
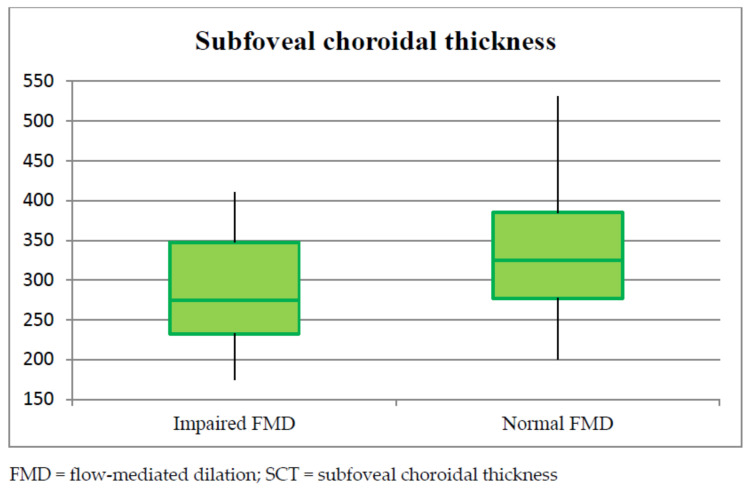
Subfoveal choroidal thickness (SCT) values in the impaired flow-mediated dilation (FMD) and normal FMD subgroups. Box plot showing the distribution of the SCT values in the impaired FMD and normal FMD subgroups.

Focusing on retinal vascular parameters, the mean RPCP-FI and RPCP-D in the total population of the study were 0.452 ± 0.017 and 0.437 ± 0.031, respectively. RPCP-FI was found to be significantly lower in impaired FMD subjects (*p* < 0.001), a difference that was observed also after binary logistic regression analysis comparing the two subgroups (*p* = 0.047). Figure 5 shows a visual representation of this result. RPCP-FI was also found to be linearly correlated with FMD values in the study population (Figure 6 and Table 2). By contrast, RPCP-D did not differ according to FMD values, neither in the dichotomous form nor in the continuous quantitative form. Nevertheless, the regression analysis showed a trend towards statistical significance of RPCP-D as concerns the FMD qualitative analysis (*p* = 0.055). Among the OCT B-scan parameters, SCT was found to be lower in patients with impaired FMD (*p* =0.004) (Figure 7), although this difference was only a trend in binary logistic regression output (*p* = 0.07) (Table 3). No linear correlation was detected between SCT values and FMD quantitative values in the total population (see Table 2). The other retinal vascular parameters evaluated in the study did not show any relevant variation between the two subgroups. Table 2 and Table 3 show data concerning the univariate analysis, logistic regression, and linear correlation of the vascular retinal parameters with FMD values. The vitreous observation revealed in 10 eyes a fibrillary retro-lens vitreous raising and no signs of inflammation. In a 58-year-old male patient, who suffered from hypertension, with a previous history of cardiac ischemia and recent coronary stent, undergoing double antiplatelet therapy (acetylsalicylic acid 100 mg and ticagrelor), an extra papillary focal retinal hemorrhage was detected. Ten (12.5%) patients showed cotton wool exudates at fundus examination.

## 4. Discussion

The continuous increase in the number of COVID-19 patient and the large number of consequent severe related complications require a global rationalization. One of the most studied complications is related to endothelial damage.

The results of our study demonstrated a higher rate of FMD impairment in early post-COVID-19 patients compared to the general population, as reported in the literature [24]. The pathogenesis of endothelial damage in COVID-19 disease is multifactorial. A rapid and well-coordinated innate immune response is the first line of defense against viral infection, whilst a dysregulated and excessive immune response may cause immune damages to the human body. In particular, the production of interferon–I (IFN-I) or IFN-α/β is the key natural immune defense response against viral infections, and IFN-I is the key molecule that plays an antiviral role in the early viral infection stage. Delayed release of IFNs in the early stages of SARS-CoV and MERS-CoV infections hinders the body’s antiviral response, causing at the same time a delayed rapid increase in cytokines and chemokines levels. This, in turn, leads to the recruitment of a massive amount of inflammatory cells which are responsible for the disruption of the tissues that stimulated their recruitment [25]. The systemic inflammatory response in COVID-19 disease, also referred to as the “cytokine storm”, highly and primarily affects the vascular endothelium. Indeed, the direct exposure to proinflammatory cytokines determines endothelial cell death and apoptosis, leading to increased vascular permeability, thromboembolism and ischemic organ damage [26]. Moreover, endothelial cells exposed to the cytokine storm initiate transcriptional programs, which induce a prothrombotic phenotype and the expression of adhesion molecules, chemokines and cytokines (such as interleukin (IL)-1 and IL-6) perpetrating the inflammatory state (proinflammatory phenotype) [27,28]. Furthermore, it is possible that in SARS-CoV-2-infected patients, antibody-dependent enhancement (ADE) of inflammation plays a role in endothelial cells’ activation and pathology [29]. Lastly, another key element in the pathogenesis of SARS-CoV-2 infection, inducing endothelial damage, is the human angiotensin-converting enzyme 2 (ACE-2). Known as the major receptor for the viral S protein, it provides the entry point for SARS-CoV-2 in a wide range of human cells, including endothelial cells and pericytes. This mechanism determines an increase in local angiotensin II levels, with consequent damage to blood vessel linings, inflammation and tissue injury [30].

According to our data, age, diabetes and RPCP-FI are independently correlated with the presence of endothelial dysfunction in the early post-infective period of SARS-CoV-2 infection. Moreover, RPCP-FI values are linearly correlated with FMD values in this population of patients. By contrast, systemic arterial hypertension and SCT, although significatively associated with impaired FMD in univariate analysis, did not relate to FMD once adjusted for other factors. Older age and diabetes are well-known risk factors for FMD impairment, which in turn predispose to cardiovascular comorbidities and major vascular events [31,32]. By contrast, the correlation of FMD with RPCP-FI is an innovative and unprecedented finding, recalling screening and diagnostic tradition connecting cardiovascular diseases and retinal findings. Different authors recently reported retinal vascular changes in COVID-19 patients. Pereira et al. reported a 22.2% prevalence of cotton wool spots and flame-shaped hemorrhages in the acute phase of the infection [33]. Similarly, Marinho et al. described subtle cotton wool spots and microhemorrhages along the retinal arcade, with no symptoms or signs of intraocular inflammation, in 4 out of 12 acute COVID-19 patients [34]. Invernizzi et al. found retinal hemorrhages in 9% of COVID-19 patients, cotton wools spots in 7% of them, dilated veins in 28% and tortuous vessels in 13% [15]. Moreover, accounting for covariates, the mean vein diameter was positively associated with COVID-19 both in severe and in non-severe cases compared with unexposed subjects and was negatively correlated with symptoms onset and positively correlated with disease severity. Nevertheless, unlike our study, these authors analyzed COVID-19 patients in the acute phase of the disease, i.e., the focus of their investigation was not on post-infective vascular sequelae associated with COVID-19 disease. Landecho et al. reported a 22% retinal microangiopathy consisting in cotton wool spots at a mean of 43 days after first COVID-19 symptoms and suggested how COVID-19 microangiopathy could represent an in vivo biomarker of systemic vascular disease [35]. The authors highlighted how cotton wool exudates are a marker of vascular disease severity in other medical contexts, such as diabetes and hypertension, and are associated with an increased risk for acute vascular events. They did not report any form of vitreoretinal inflammation such as vasculitis, hemorrhages, chorioretinal infiltrates or vitreitis, as shown for other viral retinitis. Other authors have shown a vascular impairment in ex-COVID-19 patients, reporting changes of RPCP, with decreased perfusion density and consequently lower blood supply to RNFL and decreased SCP and DCP in comparison with healthy controls. [14,36] Our study reported a 12.2% cotton wool spot prevalence in a similar cohort of post-COVID-19 patients, with no difference according to the FMD values. The finding of retinal vascular changes in COVID-19 patients needs to be carefully evaluated because of the higher prevalence of immune diseases, obesity, diabetes and cardiovascular diseases in patients manifesting a moderate to severe clinical infection course, such as those who required hospital admission. In fact, systemic cardiovascular diseases such as arterial hypertension, coronary heart disease, or diabetes mellitus, as well as obesity, are all associated with structural vascular changes in the retina. These include narrowing of arterioles, vein dilatation and a decrease in the arteriovenous ratio (AVR) [37]. The possibility to perform a regression analysis on a large patient sample is therefore of utmost importance in this context. In this perspective, it should be highlighted that our study analyzed the largest cohort of patients among all the above-mentioned studies. A limit that we could not overcome was our inability to obtain similar data from the same patients regarding the pre-infective period, for comparison. The absence of further follow-up evaluations and the impossibility to establish a cut off for the RPCP-FI values suggesting FMD impairment are among the other limits of the study. Therefore, we encourage the ophthalmic community to provide additional insight on this interesting matter in order to confirm and expand our findings.

## Figures and Tables

**Table 1:** Demographic and clinical features of the enrolled patients. Anamnestic and hospital admission variables in the total study population (82 patients), subgroup of subjects with impaired flow-mediated dilation (FMD ≤ 7%), and patients with normal values of FMD (51 patients). The last two columns on the right show the *p* value derived from univariate analysis and the *p* value derived from binary logistic regression comparing the two mentioned subgroups. The significant correlation between both demographic and clinical feature and FMD impairment was reported in bold.

Variable	Total Population (82 Pts)	Impaired FMD (31/82 Pts)	Normal FMD (51 Pts)	*p* Univariate	*p* Regression
ANAMNESTIC DATA					
Age (years)	52.9 ± 13.5	60.12 ± 11.9 (55.9–64.3)	48.2 ± 12.6 (45.0–51.9)	**<0.001**	**0.048**
Sex	M = 48/82 (58.5%)	21/31 (67.7%)	27/51(52.9%)	0.18	0.411
Systemic arterial hypertension	19/82 (23.2%)	13/31 (41.9%)	6/51 (11.76%)	**0.002**	0.417
Diabetes	36/82 (43.9%)	18/31 (58.1%)	18/51 (35.3%)	**0.04**	**0.046**
Autoimmune diseases	21/82 (25.6%)	7/31 (22.58%)	14/51 (27.45%)	0.62	0.211
BMI score	25.7 ± 4.3	25.5 ± 4.0 (24.0–26.9)	25.7 ± 4.6 (24.5–27.0)	0.78	
BMI > 30	8/82 (9.7%)	1/31 (3.2%)	7/51 (13.7%)	0.12	0.514
Chronic kidney disease	8/82 (9.8%)	5/31 (16.1%)	3/51 (5.9%)	0.08	0.324
Cognitive impairment	7/82 (8.5%)	4/31 (12.9%)	3/51 (5.9%)	0.12	0.731
ADMISSION DATA					
Hydroxychloroquine	57/82 (69.5%)	25/31 (80.6%)	32/51 (62.7%)	0.09	
Lopinavir + Ritonavir	27/82 (32.9%)	13/31 (41.9%)	14/51 (27.5%)	0.17	0.24
Darunavir + Ritonavir	35/82 (42.7%)	14/31 (45.2%)	21/51 (41.2%)	0.72	0.36
Heparin	28/82 (34.1%)	11/31 (35.5%)	17/51(33.3%)	0.84	
Azithromycin	33/82 (40.2%)	14/31 (45.2%)	14/51 (27.4%)	0.10	
Antiplatelet therapy	6/82 (7.31%)	4/31 (12.9%)	2/51 (3.9%)	0.12	
Corticosteroids	4/82 (4.87%)	1/31 (3.2%)	3/51 (5.8%)	0.59	
ICU admission	9/82 (10.9%)	5/31 (16.1%)	4/51 (7.8%)	0.62	0.581
Oxygen therapy	33/82 (40.2%)	14/31 (45.2%)	19/51 (37.3%)	0.49	0.612
Non-invasive ventilation	11/82 (13.4%)	4/31 (12.9%)	7/51 (13.7%)	0.93	0.866
Invasive ventilation	4/82 (4.9%)	2/31 (6.5%)	2/51 (3.9%)	0.76	0.643
Pulmonary embolism	2/82 (2.4%)	1/31 (3.2%)	1/51 (2%)	0.88	
Venous thrombosis	2/82 (2.4%)	2/31 (6.5%)	0/51 (0%)	0.19	

BMI = body mass index; FMD = flow-mediated dilation; ICU = intensive care unit.

**Table 2:** Analysis of the linear correlation between retinal vascular OCT and OCTA parameters and flow-mediated dilation (FMD) values in the study population. The *p*-value in bold is used to underline the linear correlation between RPCP-FI and FMD values in the study population.

Variable	Linear Correlation To FMD			*p*
RPCP-FI	R = 0.244	Slope = 81.455	Intercept = −27.467	**0.027**
RPCP-D	R = 0.212	Slope = 26.916	Intercept = −2.289	0.055
SCP-D	R = 0.116	Slope = 0.428	Intercept = 0.488	0.31
DCP-D	R = 0.110	Slope = 0.523	Intercept = 0.451	0.46
SCT	R = 0.2	Slope = 0.014	Intercept = 5.02	0.072
FAZ area	R = −0.031	Slope = −3.14	Intercept = 10.329	0.78
FAZ perimeter	R = −0.117	Slope = −1.251	Intercept = 12.16	0.31

DCP-D = deep capillary plexus density; FAZ = foveal avascular zone; FMD = flow-mediated dilation; RPCP-D = radial peripapillary capillary plexus density; RPCP-FI = radial peripapillary capillary plexus flow index; SCP-D = superficial capillary plexus density; SCT = subfoveal choroidal thickness.

**Table 3:** Retinal vascular OCT and OCTA parameters in the total study population (82 patients), the impaired flow-mediated dilation (FMD) subgroup (31 patients), and the normal FMD subgroup (51 patients). The last two columns on the right show the *p* values derived from univariate analysis and the *p* values derived from binary logistic regression comparing the two mentioned subgroups. Both univariate and binary logistic regression analysis showed that RPCP-FI was significantly lower in impaired FMD subjects (*p* value in bold). From the univariate analysis, SCT was found to be lower in patients with impaired FMD (reported in bold), while this resulted only as a trend in binary logistic regression output.

Variable	Total Population (82 Patients)	Impaired FMD (31/82 Patients)	Normal FMD (51/82 Patients)	*p* Univariate	*p* Regression
RPCP-FI	0.452 ± 0.017	0.445 ± 0.019 (0.439–0.452)	0.458 ± 0.014 (0.455–0.462)	**<0.001**	**0.047**
RPCP-D	0.437 ± 0.031	0.432 ± 0.037 (0.419–0.445)	0.441 ± 0.027 (0.433–0.448)	0.21	0.055
SCP-D	21.27 ± 1.32	21.05 ± 1.50 (20.50–21.59)	21.40 ± 1.22 (21.07–21.74)	0.25	0.31
SCP-P	0.385 ± 0.021	0.371 ± 0.08 (0.380–0.369)	0.390 ± 0.12 (0.396–0.377)	0.45	0.63
DCP-D	21.82 ± 2.51	21.96 ± 2.38 (21.44–22.23)	21.56 ± 2.52 (21.24–21.73)	0.37	0.43
DCP-P	0.456 ± 0.03	0.453 ± 0.05 (CI 0.448–0.460)	0.459 ± 0.01 (CI 0.453–0.465)	0.86	0.77
SCT	310.46 ± 43.13	278.45 ± 79.36 (250.51–306.39)	329.92 ± 47.38 (308.69–351.16)	**0.004**	0.07
FAZ area	0.237 ± 0.106	0.236 ± 0.099 (0.200–0.271)	0.238 ± 0.112 (0.207–0.269)	0.91	0.85
FAZ perimeter	2.058 ± 0.506	2.082 ± 0.427 (1.926–2.237)	2.045 ± 0.553 (1.892–2.199)	0.76	0.76
Cotton wool spots	10/82 (12.2%)	4/31 (12.9%)	6/51 (11.8%)	0.32	0.51

DCP-D = deep capillary plexus density; DCP-P = deep capillary plexus perfusion; FAZ = foveal avascular zone; FMD = flow-mediated dilation; RPCP-D = radial peripapillary capillary plexus density; RPCP-FI = radial peripapillary capillary plexus flow index; SCP-D = superficial capillary plexus density; SCP-P = superficial capillary plexus perfusion; SCT = subfoveal choroidal thickness.

## Data Availability

The datasets generated and/or analyzed during the current study are available from the corresponding author on a reasonable request.
